# Long-term recurrence and complication rates after incisional hernia repair with the open onlay technique

**DOI:** 10.1186/1471-2482-9-6

**Published:** 2009-04-28

**Authors:** Lars Peter Holst Andersen, Mads Klein, Ismail Gögenur, Jacob Rosenberg

**Affiliations:** 1 Dept. of Surgery D, Herlev Hospital, University of Copenhagen, 2730 Herlev, Denmark

## Abstract

**Background:**

Incisional hernia after abdominal surgery is a well-known complication. Controversy still exists with respect to the choice of hernia repair technique. The objective of this study was to evaluate the long-term recurrence rate as well as surgical complications in a consecutive group of patients undergoing open repair using an onlay mesh technique.

**Methods:**

Consecutive patients undergoing open incisional hernia repair with onlay-technique between 01/05/1995 and 01/09/2007 at a single institution were included in the study. For follow-up patients were contacted by telephone, and answered a questionnaire containing questions related to the primary operation, the hernia and general risk factors. Patients were examined by a consultant surgeon in the outpatient clinic or in the patient's home if there was suspicion of an incisional hernia recurrence.

**Results:**

The study included 56 patients with 100% follow-up. The median follow-up was 35 months (range 4–151). Recurrent incisional hernia was found in 8 of 56 patients (15%, 95% CI: 6–24). The overall complication rate was 13% (95% CI, 4–22). All complications were minor and needed no hospital admission.

**Conclusion:**

This study with a long follow-up showed low recurrence and complication rates in patients undergoing incisional hernia repair with the open onlay technique.

## Background

Incisional hernia is a well-known complication after abdominal surgery, with incidence rates of approximately 3% and 15% after laparoscopic and open surgery, respectively [1]. Hernias are associated with reduced quality of life and high socioeconomic costs [2]. The treatment of incisional hernias have changed radically over the last decade, however controversy still exists concerning mesh type [3], mesh positioning [4] and operation method, with laparoscopic repair as an increasingly preferred alternative to open surgery [5]. In the present study we evaluated the long-term recurrence and complication rates after incisional hernia repair with open onlay technique in a consecutive series of patients.

## Methods

**Figure 1:**
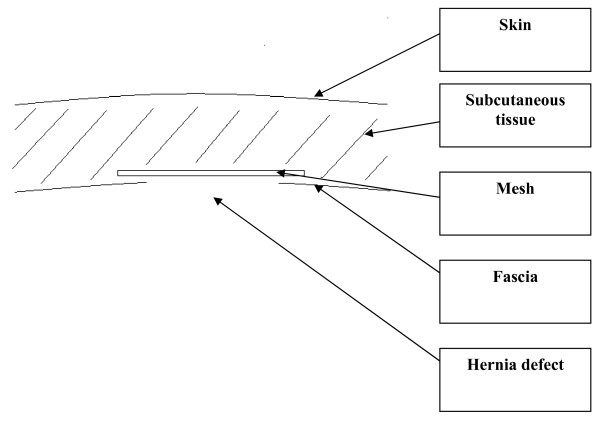
**Schematic drawing of the mesh position**. The hernia defect was routinely sutured and the onlay mesh was used as a reinforcement of the suture line.

The study included consecutive patients who underwent open hernia repair with onlay technique between May 1995 and September 2007 (see figure 1). All hernias disregarding size were operated by the same technique which included closure of the hernia defect with non-absorbable sutures (typically Prolene 2-0) followed by an onlay polypropylene mesh (figure 1). Thus, the mesh was placed superficial to the external fascia also using non-absorbable sutures. Repair operations were carried out by four senior surgeons at a single institution.

Each patient was contacted by telephone and underwent a structured interview. The interview contained questions concerning previous operations for incisional hernia, suspected or diagnosed incisional hernia, and other types of hernia (e.g. inguinal). All patients were interviewed between January 2008 and August 2008. A recurrence was "suspected" if the patient was not sure if he/she had a hernia or not. A hernia was "diagnosed" if the primary care physician or a surgical specialist had examined the patient and established the diagnosis. In the interview, hernias were described regarding reducibility, aggravating factors, cosmetic inconvenience, and mental stress related to the hernia. Wound length, localisation of the incision, wound complications and type of treatment were registered as well as patient related risk factors such as co-morbidity, use of systemic corticosteroids, and use of alcohol or tobacco. If the patient had a suspected hernia or a diagnosed hernia he was examined by a single consultant surgeon in the outpatient clinic or in the patient's home.

Data are reported as frequencies (CI – 95% confidence intervals) and median (range) unless stated otherwise. For analysis of categorical and continuous data, Fishers exact test and Mann Whitney's test were used. P < 0.05 was considered statistically significant. The study was approved by the local ethics committee.

## Results

**Figure 2:**
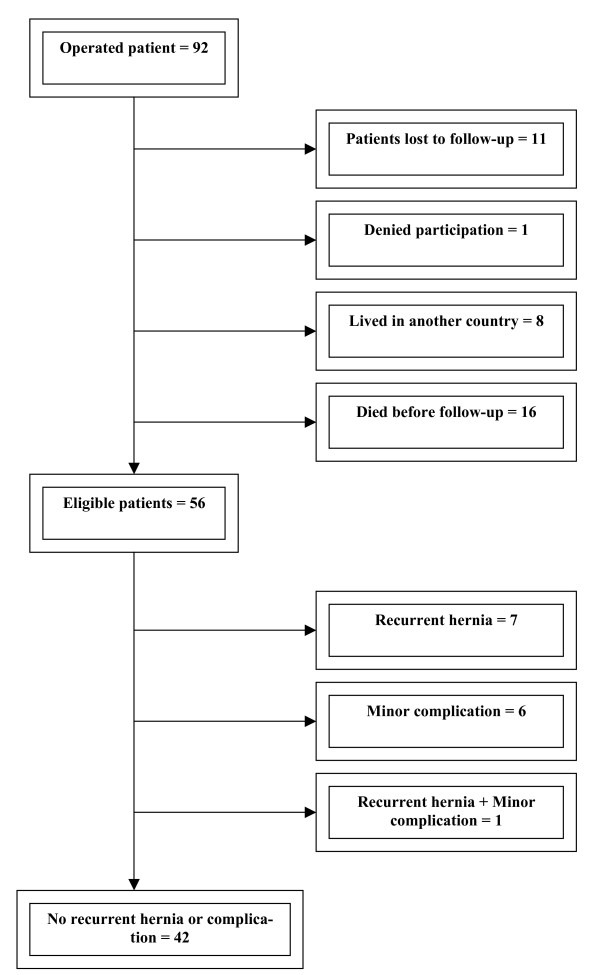
**Flow chart of the study cohort**.

A total of 92 patients had an incisional hernia repair with open onlay technique during the inclusion period. However, 36 patients could not participate in the study for various reasons (Figure 2). Thus, the study included 56 patients with 100% follow-up.

Demographics (age, gender, weight, alcohol, tobacco), risk factors (prostate hypertrophy, diabetes mellitus, chronic obstructive pulmonary disease, the number of self reported co-morbidities), the length of the follow-up period, and hernia related data are presented in table 1. All demographic data are at the time of follow-up.

**Table 1:** Demographic data in patients with or without recurrent hernia

**Variable**	**No hernia (n = 48)**	**Hernia (n = 8)**
Age (years)	62 (29–92)	76 (50–86)

Gender (M/F)	23/25	3/5

BMI (kg/m2)	26 (18–44)	26 (21–31)

Length of incision (cm)	12 (3–27)	8.5 (3–20)

Chronic lung disease	10	1

Constipation	15	4

Diabetes	3	0

Smoking	18	3

Alcohol abuse	3	2

Systemic corticosteroid treatment	1	1

None of the demographic data or risk factors were statistically significant between groups.

The median observation time was 35 months (range 4–151). In our follow-up we found by clinical examination that 4 of the 56 patients had developed a hernia. Another 4 patients had undergone a repair operation for a recurrent incisional hernia in the follow-up period before the interview making the total number of recurrences 8 of 56 patients (15%, 95% CI: 6–24).

An overall complication rate of 13% (95% CI, 4–22) was found. Three patients were treated for seroma or haematoma with evacuation of fluid. One patient was treated for wound infection with antibiotics. One patient had a secondary closure of the wound due to exudation, and one patient had a minor complication, but was unable to remember the specific treatment at follow up. None of these patients had a recurrent hernia on follow-up. One patient was diagnosed with seroma/hematoma but received no treatment, however, this patient later developed a hernia. None of these complications required hospital admission, thus there were no serious complications after the hernia repair.

## Discussion

In this study we have shown a low long-term recurrence rate of 15% and an overall rate of non-serious complications of 13% after open hernia repair using the onlay mesh technique in a single institution.

The surgical treatment of incisional hernia has changed rapidly during the last decade with the increasing use of mesh technique and the introduction of laparoscopy. However, many questions concerning mesh type, mesh positioning, fixation method and operation type still remain unanswered [3,4]. Patients with incisional hernia are a heterogeneous population with patient-specific co-morbidity and innate differences (e.g. collagen formation quality) [6]. This makes the choice of technique most suitable for each patient even more difficult.

A recent retrospective study including 161 patients comparing open suture technique with open onlay technique found recurrence rates of 31% versus 11%(p < 0.05) [7]. The onlay technique was superior with respect to return to physical activity. Patients operated with the onlay technique had a significantly higher complication rate (seroma 42% vs. 8%, suppuration 16% vs. 10%) and longer hospitalization time (mean 13 vs. 9 days). The conclusions of the study were limited by a patient number of only 28 in the onlay group.

In a randomized clinical trial by Korenkov et al. comparing suture repair, onlay mesh technique and autodermal graft, the authors found that onlay technique was associated with a higher serious infection risk and significantly higher pain scores [4]. The study was ended prematurely because of the rate of serious complications. The higher infection rate could not be explained by the authors.

A retrospective study by de Vries Reilingh et al. compared mesh repair with inlay, onlay and sublay technique, and concluded that sublay was superior to the other techniques [8]. Onlay technique had a significantly higher wound complication rate (69%) compared to the inlay (13%) and sublay (12%) technique. Three patients in the onlay group developed reherniation (23%). This study only included 13 patients with onlay technique and had a mean follow-up of only 19 months.

Kingsnorth et al. found a recurrence rate of 3.4% after a 15.2 month follow up period using the onlay technique [9]. Post operatively 11 patients (9.5%) developed seroma and 2 patients (1.7%) had a deep wound infection. The study was a prospective audit including 116 patients.

Our study included 56 consecutive patients in a single institution with a median follow-up period of 35 months (range 4–151). In accordance with previous studies we chose to combine a structured interview with a clinical examination [1,10-13]. This method proved to be effective with a clinical examination of all eligible patients suspected of hernia. Our study group showed no significant differences with respect to distribution of known risk factor between patients with or without recurrent hernia.

The onlay technique is a simple and effective repair operation with a short learning period for the surgeon. For open incisional hernia repair the choice between inlay, onlay and sublay technique is often based on tradition and the individual surgeon's expertise rather than scientific evidence. It has been routine to perform all incisional hernias by the onlay technique at the institution involved in the present study, and we therefore don't have any patients who had been operated by other open techniques. Our study shows that the onlay technique seems to be safe in terms of complication and recurrence rates for the patient. In addition this technique requires little tissue dissection with an easy access to the hernia repair. These advantages should be taken into consideration when choosing between laparoscopic and open technique and when choosing between different open technique. The laparoscopic approach is generally associated with at longer learning curve.

The present study has several limitations. It is retrospective and has a limited number of patients. Twenty operated patients did not participate, and 16 patients had died before follow-up. This is a consequence of the long follow-up period. In addition many of the patients lived outside the country and could not be contacted to participate in the follow-up.

The introduction of laparoscopic technique is an increasingly used alternative to open surgery. A prospective randomized study by Olmi et al. compared the short-term outcome of laparoscopic versus open repair technique [14]. The authors found significantly shorter operative time, shorter hospitalization, lower complication rate and faster return to work when using laparoscopic technique. There was a total complication rate of 16% among patients undergoing laparoscopic repair, and 29% among patient undergoing open surgery. The study included 170 patients and patients were followed for a median of 24 months. The hernia recurrence rate was 2.3% and 1.1% in the laparoscopic and open group, respectively.

In a recent systematic review by Müller-Riemenschneider et al., evaluating the long term prognosis, a trend towards lower recurrence rates, shorter hospital stay, lower complication rates, and lower pain scores after laparoscopic approach was found, but with a higher frequency of intestinal perforation [5]. However, all identified studies suffered from significant methological limitations such as different baseline characteristics among patients, short follow-up periods or small patient numbers, and long-term results after laparoscopic repair still remains unknown. Our results, although from a single institution, show very low and clinical insignificant complication rates and a very low long-term recurrence rate making the open onlay mesh technique a serious alternative to laparoscopic repair in selected patients. Future large scale randomized studies should clarify indications for laparoscopic versus open onlay technique.

## Conclusion

We found low long term recurrence and complication rates after open hernia repair with onlay mesh technique. These results require confirmation by randomized clinical trials.

## Competing interests

In the past five years JR has received research grants from Karl Storz, Johnson & Johnson and Covidien for other studies. The present study was not supported financially in any way.

## Authors' contributions

All authors participated in the design of the study and the acquisition of data. The manuscript was drafted by LPHA with critical revisions by IG and JR. All authors read and approved the final manuscript.

## Pre-publication history

The pre-publication history for this paper can be accessed here:


